# Patellar Dislocation Masking a Tibial Post Fracture: An Unusual Complication of Posterior Stabilized Total Knee Arthroplasty

**DOI:** 10.7759/cureus.48950

**Published:** 2023-11-17

**Authors:** Aditya Seth, Pranav K Buddhapuram, Arvind Kadwad, Sandeep Boddeda, Ratnakar Vecham, Adarsh Annapareddy, A.V. Gurava Reddy

**Affiliations:** 1 Department of Orthopedics, KIMS-Sunshine Hospital, Hyderabad, IND

**Keywords:** posterior stabilized knee, cam and post, polyethylene post breakage, arthroplasty complication, chronic patella dislocation, arthroplasty, tibial post fracture

## Abstract

Posterior stabilized total knee arthroplasty (TKA) has established itself as a highly effective design for total knee arthroplasty, renowned for its longevity and success. However, a subset of cases, approximately 6-12%, faces early failure, necessitating revision procedures. This case report presents a unique and previously undocumented complication involving a tibial post fracture following hyperflexion of the knee, masked by chronic patellar dislocation. This case highlights the importance of considering polyethylene wear-related failure in cases of instability without an apparent history of trauma. The surgical intervention involved polyethylene insert exchange, patellar debulking, lateral retinacular release, and quadriceps tendon double-breasting.

## Introduction

The posterior stabilized total knee arthroplasty (TKA) implant stands out as one of the remarkably successful designs within the realm of total knee arthroplasty, and its efficacy is well-documented in the literature [1-3]. Over time, it has garnered recognition as the gold standard for ensuring the longevity of TKA procedures [2].

The foundation of this design lies in its role as a posterior cruciate ligament-substituting implant, where the native posterior cruciate ligament (PCL) is effectively replaced by a tibial post and femoral cam mechanism [3,4]. This construct facilitates femoral roll-back during knee flexion while effectively averting posterior tibial subluxation throughout the flexion process [4].

Nevertheless, it is crucial to recognize that despite the overall success of primary TKAs, a notable subset of cases, approximately 6-12%, encounters failure within the first decade post-surgery, necessitating revision procedures [5]. Remarkably, there is limited literature addressing polyethylene insert fracture being masked by patellar subluxation and instability.

In the present case, we draw attention to an exceptionally rare complication; the occurrence of a tibial post fracture following hyperflexion of the knee masquerading as patellar dislocation, several years after the patient's initial posterior stabilized total knee arthroplasty. To the best of our knowledge, this specific presentation and fracture concurrently has not been previously documented in the existing medical literature.

## Case presentation

**Figure 1:**
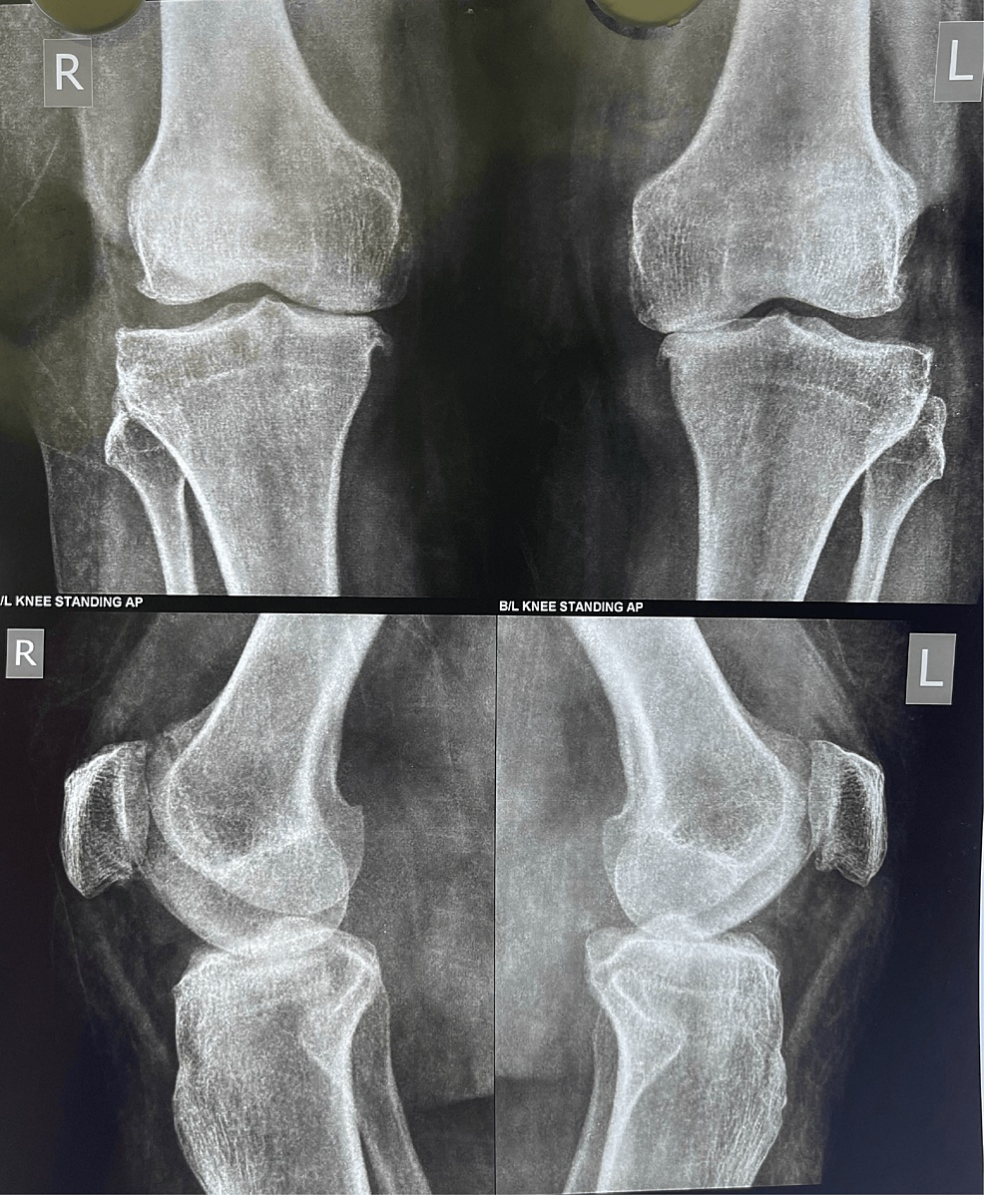
Preoperative radiograph showing a varus deformity with severe osteoarthritis over the medial side in both knees

**Figure 2:**
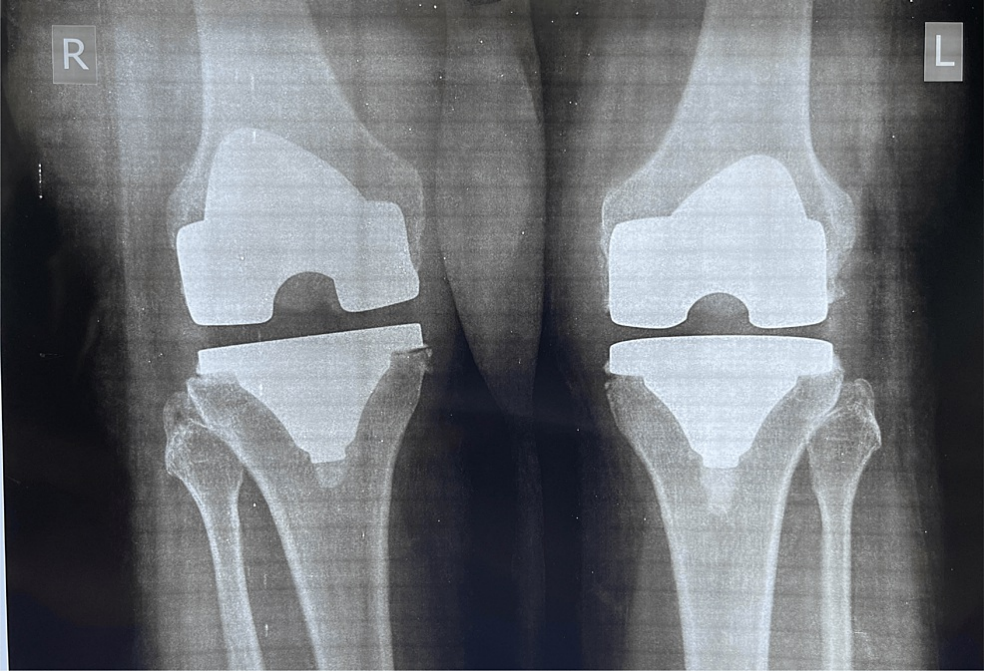
Three months postoperative weight-bearing radiograph showing well-balanced knees

A 61-year-old female patient presented to our outpatient department (OPD) with complaints of a clicking sensation in her right knee and a feeling of instability. She had undergone bilateral simultaneous total knee replacement surgery with Stryker NRG implants (Stryker Orthopedics, Kalamazoo, MI, USA) approximately eight years prior. Figure 1 shows the patient's preoperative radiograph and Figure 2 shows the three-month postoperative radiograph.

The patient's medical history was devoid of any traumatic events. She did, however, recount an incident where she hyperflexed her right knee during a religious ritual. This event was followed by pronounced swelling, pain, and a temporary inability to bear weight on the affected leg. During our initial consultation, the patient did not mention any auditory phenomena associated with the hyperflexion incident. However, upon subsequent probing after surgery, she revealed that she had heard a distinct audible pop sound during the episode.

**Figure 3:**
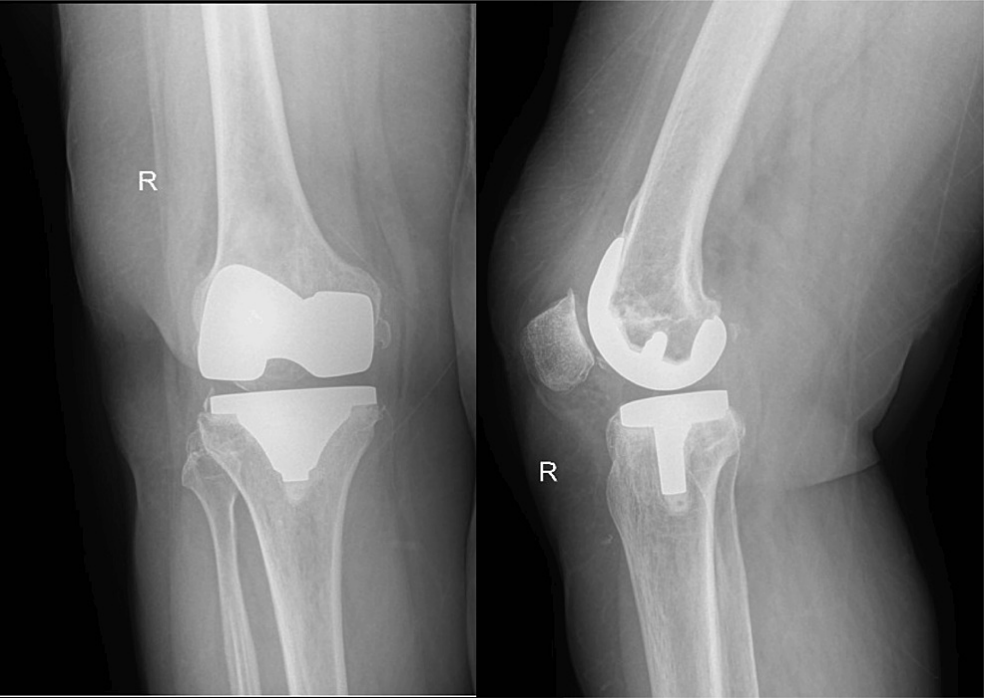
The anteroposterior and lateral views of the knee in the standing position These radiographs show a well-balanced knee; however, the patella position couldn't be commented on in these radiographs.

**Figure 4:**
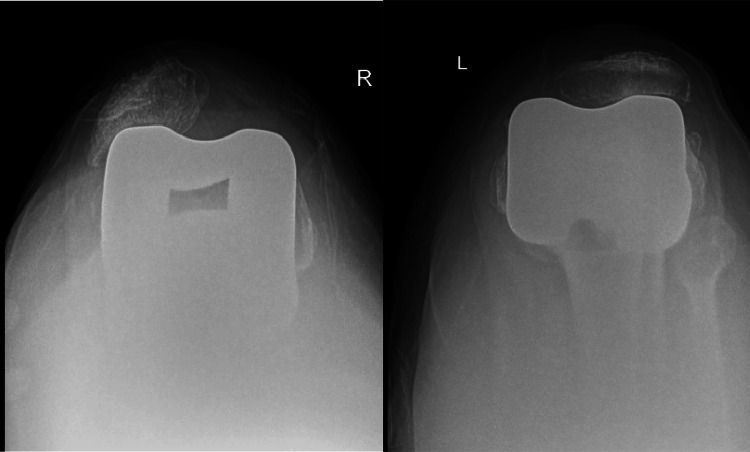
Skyline view of the knee with a patellar dislocation on the right side

Upon initial examination, the patient's patella exhibited dislocation on the right side. The evaluation revealed grade 2 instability in the anteroposterior plane, with no evidence of varus-valgus instability. Radiographs were obtained, which confirmed the finding as shown in Figures 3, 4.

The patient exhibited no clinical signs of infection, as indicated by her blood parameters within the normal range (erythrocyte sedimentation rate (ESR) - 16 mm/hr, C-reactive protein (CRP) - 0.30 mg/dL, total leucocyte count (TLC) - 6,600 cells/cumm).

In the course of the clinical assessment, the clicking sounds were linked to the patellar dislocation, which was also evident in the radiographs taken at the time of presentation. This prompted the decision to schedule a surgical procedure for patellar realignment.

**Figure 5:**
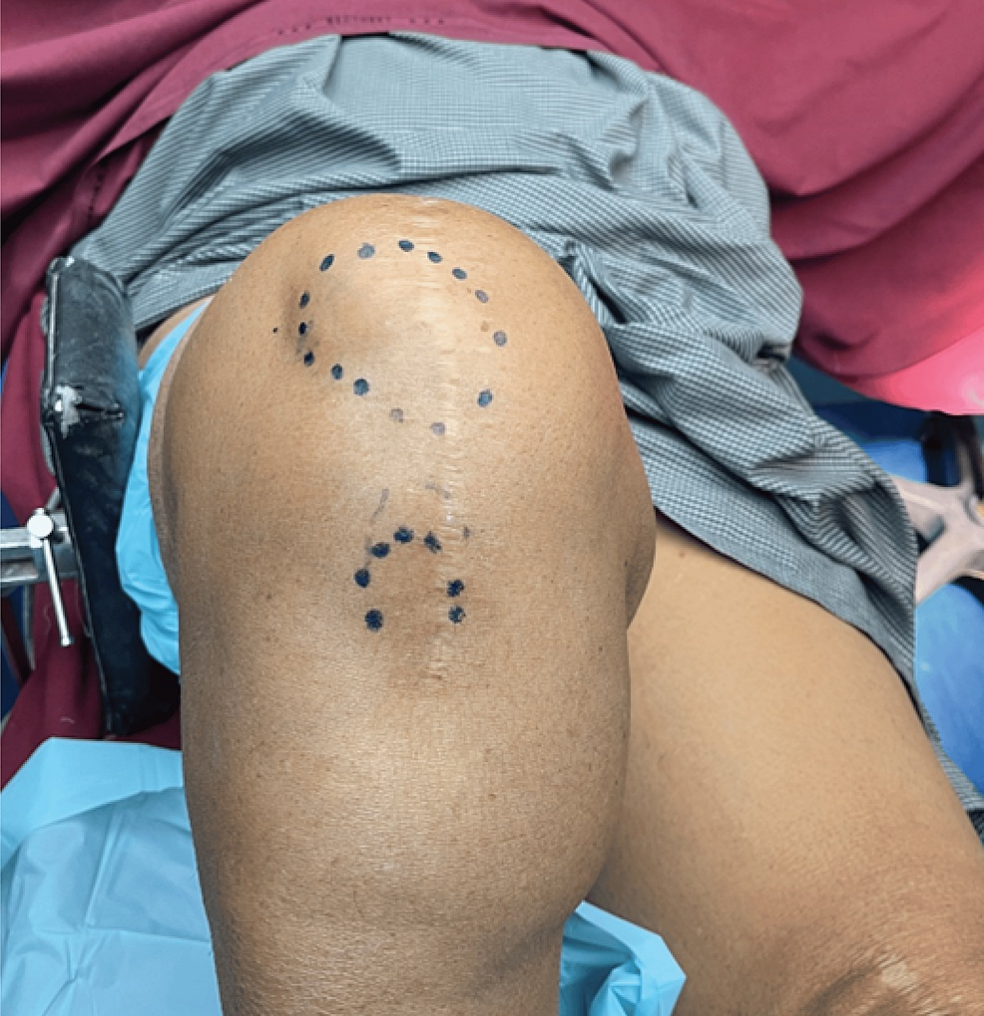
Dislocation of the patella upon flexion of the knee after the administration of anesthesia

Following the administration of anesthesia, flexion of the knee patellar dislocation was evident as shown in Figure 5.

**Figure 6:**
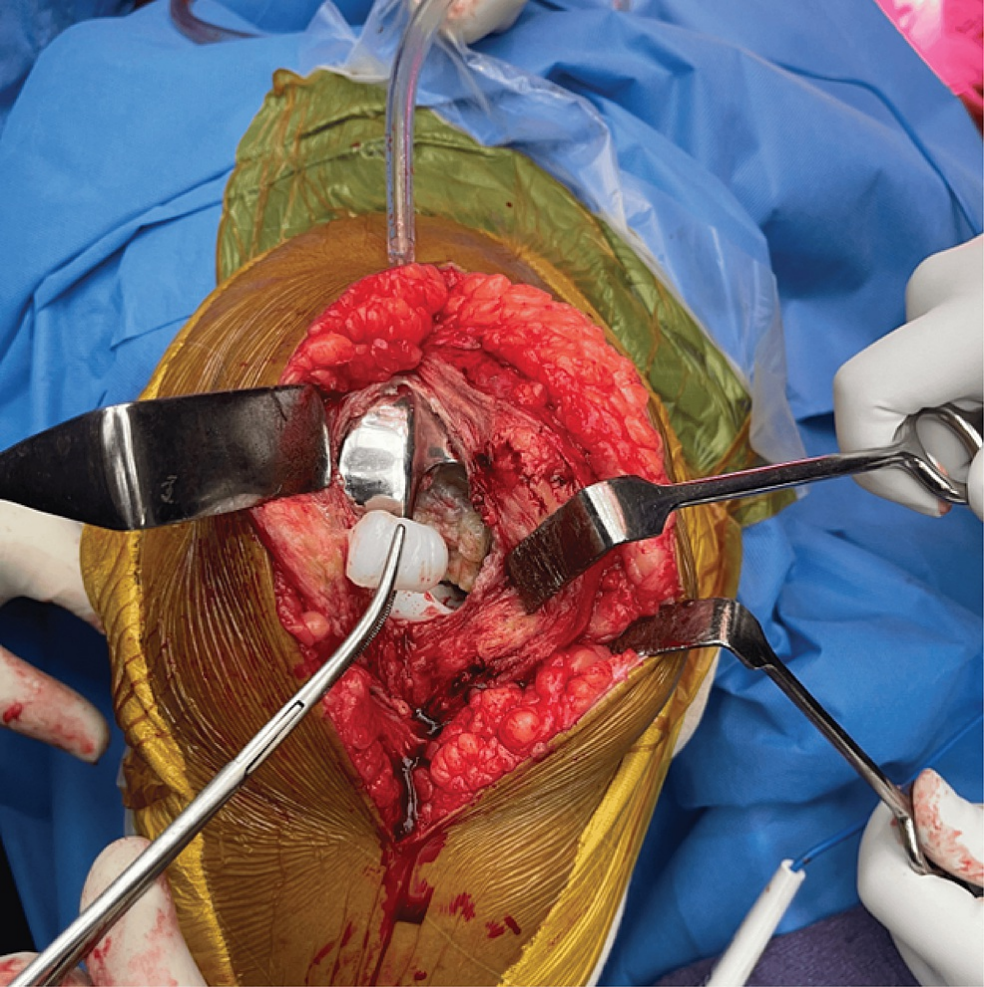
Tibial post fracture

However, upon conducting an arthrotomy, an unforeseen complication emerged, as the tibial post was identified as fractured, as seen in Figure 6, concomitant with an increase in the flexion and extension gaps.

The polyethylene insert was replaced with a larger 15mm size from 12mm due to an increase in both flexion and extension gaps. Additionally, debulking of the patella along with the removal of osteophytes followed by lateral retinaculum release and double-breasting technique was employed for the quadriceps tendon.

**Figure 7:**
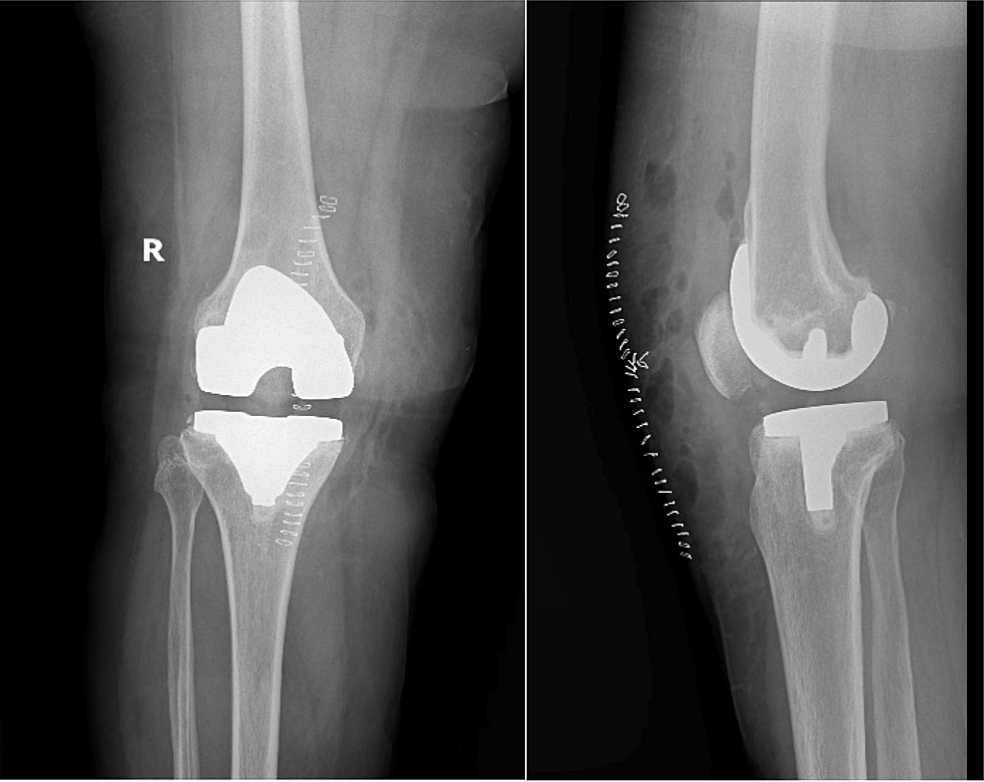
Postoperative radiograph of the right knee in the supine position

Subsequent to the surgical procedure, a radiograph was taken postoperatively as shown in Figure 7 and the patient received post-operative instructions, advising a range of motion of up to 30 degrees, with the prescription of a long knee brace to facilitate full weight-bearing. This comprehensive approach aimed at restoring the patient's knee functionality and mitigating her symptoms effectively.

## Discussion

In our presented case, a significant observation was the chronic patella dislocation on the patient's right side and subluxation of the patella on the left side. This is further corroborated by the evident contouring of the lateral facet, which, when analyzed in conjunction with the femoral component on the skyline view, offers a clear indication of the dislocation's chronic nature as shown in Figure 3. Such findings emphasize the importance of thorough radiographic examinations in assessing the nuances of knee pathologies and understanding their potential implications.

Patellar stability is contingent upon several critical factors, including precise implant positioning, optimal soft-tissue balance, accurate bone resections, and the design of femoral and patellar components that are conducive to patellar health. Among these considerations, the design of femoral components assumes particular significance. Femoral components characterized by a shallow and symmetric trochlear groove, featuring abrupt changes in sagittal radius, have the potential to disrupt normal patellar kinematics and heighten the risk of patellar maltracking [6-8].

Surgical inaccuracies frequently underlie issues related to patellar instability. These inaccuracies encompass residual valgus limb malalignment, patella alta (an elevated patella), an increased internal rotation of either the femoral or tibial component, medial translation of the femoral component, excessive valgus alignment of the femoral component, asymmetric patellar resection, lateral positioning of the patellar button, and excessive patellar thickness [7-9]. These factors collectively play a substantial role in patellar stability and underscore the significance of precision in implant design and surgical execution.

Previous studies have extensively documented the occurrence of wear and failure of the tibial post, as presented in Table 1 [10]. The literature suggests a multitude of factors [11-15] that contribute to the wear and erosion of the tibial post. One such factor includes the increased posterior slope of the tibial baseplate, which may lead to premature and less-than-ideal contact between the femoral component and the post. Additionally, the rotation of the femoral component has been identified as a potential contributor to tibial post failure, particularly when it results in edge loading on non-rounded posts and cams. Another aspect to consider is the excessive flexion of the femoral component, which may precipitate cam post-impingement.

Furthermore, the wear of the tibial post is influenced by dynamic physiological conditions, specifically the velocity at which the post-cam engagement occurs and the angle of flexion during engagement [16]. In the most severe instances, complete erosion of the tibial post has been observed. Moreover, although infrequent, a significant complication that has been noted is the fracture of the tibial post. Such fractures are rare, occurring in less than 1% of cases, but they are often associated with wear and deformation of the post [17-20].

**Table 1:** Tibial post complications reported in the literature in posterior-stabilized total knee arthroplasty

Study	Year	Mode of Failure	Diagnosis	Comments
Ip et al. [21]	2004	Atraumatic	Patella clunk syndrome	The IB II prosthesis (PS design) is especially prone to cause patella clunk syndrome
Jung et al. [22]	2009	Atraumatic	Tibial post-fracture	Hard mass palpable in the suprapatellar pouch
Kumar et al. [18]	2015	Atraumatic	Tibial post-fracture and polywear	Complaint of pain and swelling, locking, and clicking sensation
Lim et al. [19]	2009	Traumatic	Tibial post-fracture and polywear	Pain, effusion, genu recurvatum, patella fracture, and soft endpoints on varus-valgus stress test
Bal et al. [4]	2008	Traumatic/Atraumatic	Tibial post-delamination	Excessive femoral component flexion, anterior positioning of the tibial tray, excessive posterior tibial slope, and joint line alterations of 8 mm or greater can predispose to anterior tibial post-impingement and failure
Clarke et al. [23]	2004	Traumatic	Tibial post-fracture	Calf pain, swelling, locking sensation

As depicted in the case report, it becomes evident that tibial post failure following TKA can occur in the absence of any obvious history of trauma or falls. In our case, it happened after hyperflexion of the knee. However, in our case, the evident patellar dislocation masqueraded as the tibial post fracture. To the best of our knowledge, no such case report has been previously reported in the literature.

When confronted with cases of instability and chronic patellar dislocation in the absence of documented trauma, it is incumbent upon surgeons to consider the potential contribution of polyethylene wear-related failure.

## Conclusions

In summary, this case report sheds light on an exceptional and rarely encountered scenario in the realm of knee arthroplasty. The occurrence of a tibial post fracture, initially concealed by chronic patellar dislocation, presents a diagnostic challenge. Such a presentation, with concurrent patellar instability and tibial post fracture, has not been previously documented in medical literature. Surgeons should be alert to the possibility of polyethylene wear-related failure in cases of unexplained instability, even in the absence of apparent traumatic events. Timely recognition and appropriate intervention can lead to successful outcomes and improved patient quality of life. This case serves as a reminder of the complexities that can arise in knee arthroplasty and highlights the need for comprehensive evaluation and meticulous surgical management when faced with atypical clinical presentations.
